# Downregulation of NDR1 contributes to metastasis of prostate cancer cells via activating epithelial‐mesenchymal transition

**DOI:** 10.1002/cam4.1532

**Published:** 2018-05-07

**Authors:** Juntao Yue, Huimin Sun, Shijie Liu, Fei Yu, Shanshan Wang, Fuli Wang, Ruixiong Shen, Feng Zhu, Lei Zhang, Chen Shao

**Affiliations:** ^1^ Department of Urology Xijing Hospital The Fourth Military Medical University Xian China; ^2^ Department of Urinary Surgery Xiangan Hospital Xiamen University Xiamen China; ^3^ Department of Urology Zhongshan Hospital Xiamen University Xiamen China; ^4^ Department of Biochemistry and Molecular Biology School of Basic Medicine Huazhong University of Science and Technology Wuhan China; ^5^ Department of Epidemiology Faculty of Preventive Medicine The Fourth Military Medical University Xian China

**Keywords:** epithelial‐mesenchymal transition, metastasis, NDR1, prostate cancer

## Abstract

The 5‐year survival rate decreases rapidly once the prostate cancer has invaded distant organs, although patients with localized prostate cancer have a good prognosis. In recent years, increasing numbers of reports showed that circulating tumor cells (CTCs) may play an important role in tumor metastasis and they have stronger potential of invasion and migration compared with their parental cells. In our previous investigation, we isolated CTCs from prostate cancer cell lines PC3. In this study, we found a novel antimetastasis gene NDR1 by analyzing different gene expression between CTCs and PC3. Lower NDR1 gene and protein expression were found in both prostate cancer cell lines and clinical specimens. Besides, NDR1 function acting as metastasis inhibitor was discovered both in vitro and in vivo. Further, we also discovered that several epithelial‐mesenchymal transition (EMT)‐related genes were upregulated when decreased NDR1 in PC3 cell lines. Therefore, our results revealed a role of NDR1 in the suppression of prostate cancer cell metastasis and provided a potential mechanism of action, thus offering new therapeutic strategies against prostate cancer metastasis.

## 1 INTRODUCTION

As the first commonest diagnosed cancer and the fifth cancer‐related mortality in men, prostate cancer has become an increasingly serious public health problem. In 2015, there were 1618 000 incident cases of prostate cancer and 366 000 deaths.^1^ Moreover, significant epidemiological differences have been observed in different areas, races, and economic conditions.^2^, ^3^ For patients with localized prostate cancer, radical prostatectomy is a standard treatment and has a good curative effect.^4^ However, metastases are present in 35% of prostate cancer patients 5 and even in 40% of patients at the time of diagnosis.^6^ In addition, the 5‐year survival rate drops from 99% to 28% once the local disease become metastatic.^3^ Therefore, elucidating the process triggering to metastasis and finding key factors specifically involved in metastatic prostate cancer may be useful to improve survival rate of prostate cancer patients at late stage.

NDR1, also known as serine/threonine kinase 38 or STK38, belongs to NDR (nuclear Dbf2‐related) family of kinases, which has been found in many species, including yeast, drosophila, and mammals.^7^ In humans, the NDR family of kinases are usually considered to regulate cell mitosis, embryonic development, centrosome duplication, and size of organs.^7^, ^8^ However, the role of NDR1 in carcinogenesis, cancer cell migration, and invasion remains unclear and ambiguous. Some studies indicate that NDR1 acts as a proto‐oncogene in progressive ductal carcinoma in situ,^9^ lung adenocarcinoma,^10^, ^11^ and ovarian cancer.^12^ On the contrary, NDR1 mRNA has been reported downregulated in samples of patients suffering from prostate cancer.^13^, ^14^, ^15^, ^16^ Likewise, some animal experiments suggest that mammalian NDR1 has a role as a tumor suppressor protein (Cornils, H., Stegert, M.R., Dirnhofer, S., and Hemmings, B.A., unpublished data).^17^ Recent studies reveal that NDR1 is a member of the mammalian Hippo pathway,^18^ which usually been known as a tumor suppressor pathway.^19^

Epithelial‐mesenchymal transition (EMT) has a key role in cancer metastasis, as epithelial cells lose their typical characteristics acquiring motile mesenchymal features.^20^ Multiple EMT‐related factors are indispensable for metastasis development. Actually, the replacement of some epithelial markers (like E‐cadherin) by mesenchymal markers (Snail, Slug, Twist1, Zeb1/2) during prostate cancer formation and metastasis has already been reported.^21^, ^22^, ^23^, ^24^ As an important process of cancer metastasis, EMT has more or less relationship with various signaling pathway, such as Wnt, TGF‐β, Notch, and Hippo signal pathway.^25^, ^26^, ^27^, ^28^ Therefore, we speculate that NDR1 may play a role in prostate cancer progression by affecting on EMT.

In this study, several genes in human prostate cancer cell line PC3 and circulating tumor cells (which were isolated and cultured as explained in our previous work^29^) were analyzed by Gene Chip, revealing NDR1 kinase as a novel key factor in the metastasis of prostate cancer. Additionally, we also give some evidence to confirm that NDR1 might be a prognostic marker for prostate tumor metastasis.

## 2 MATERIALS AND METHODS

### 2.1 Cells

PC3 and HEK293T cells cell line were purchased from the American Type Culture Collection (ATCC, Rockville, MD, USA) and cultured separately in F12‐K or DMEM medium supplemented with 10% fetal bovine serum (FBS). LNCaP and C4‐2 cell lines were kindly provided by Dr Chung (Cedars‐Sinai Medical Center, CA, USA) and cultured in RPMI 1640 medium supplemented with 10% FBS. The C4‐2 cell line was derived from subcutaneous xenograft tumor of LNCaP in nude mice.^30^ Compared with LNCap cell, the C4‐2 cell has stronger metastatic ability. CTC cells were isolated and cultured from blood of nude mice with PC3 cell injected in prostate. Besides, the metastatic capacity between CTC and PC3 cell was compared, and results showed CTC was more invasive than PC3 whether in vivo or in vitro.^29^ All the cell lines above‐mentioned were incubated at 37°C in a 5% CO_2_ incubator.

### 2.2 Antibodies

The primary antibodies used in this study were the following: NDR1 mouse monoclonal antibody (1:500 dilution) was purchased from Santa Cruz Biotechnology (CA, USA), MMP‐2 rabbit monoclonal antibody (1:1000 dilution), MMP‐9 rabbit monoclonal antibody (1:1000 dilution), human EMT Antibody Sampler Kit including nine EMT‐related primary antibodies (1:1000 dilution) were purchased from Cell Signaling Technology (Beverly, MA, USA).

### 2.3 RT‐PCR

Total cell RNA was extracted from 4 cells using TRIZOL reagent (Takara, Japan). cDNA synthesis was performed by PrimeScript^™^ RT reagent Kit with gDNA Eraser (Takara, Japan). qPCR was performed using SYBR^®^ Premix Ex Taq^™^ II (Takara, Japan), and fluorescence was detected using 7500 Fast real‐time instrument (Applied Biosystems, CA, USA). Experiments were repeated 3 times and data were analyzed using the 2^−ΔΔCt^ method, using GAPDH as endogenous control. PCR primer sequences were as follows: NDR1 forward sequences, CGCAATTGCAATGACAGGCTCAACACC; NDR1 reverse sequences, GCCTCGAGCTATTTTGCTGCTTTCATGTAGG; GAPDH forward sequences, CACCCAGAAGACTGTGGATGGC; GAPDH reverse sequences, GTTCAGCTCAGGGATGACCTTGC.

### 2.4 Western blot analysis

Cells were seeded in 10‐cm dishes and were collected and lysed using 300 μL RIPA buffer containing 1% protease inhibitor when cells were adherent to the bottom of the dishes and confluent at a density of approximately 5 × 10^7^. The lysate was stored on ice for 20 minutes and sonicated 3 times for 15 seconds each time, then centrifuged at 9500 g for 15 minutes at 4°C. BCA assay method was performed for detecting total protein concentration. Loading buffer 5× SDS was added to the sample and boiled for 10 minutes before loading into 10% SDS polyacrylamide gel. Next, the protein was transferred on PVDF transfer membrane (Millipore, Billerica, MA, USA), and the membrane was incubated overnight with primary antibodies. ECL method was used for detecting chemiluminescence, and digital imaging was obtained by ChemiDoc MP Imaging System (BIO‐RAD, CA, USA). Signal quantification of each band was measured by Image J software (Sun Microsystems, Inc, CA, USA).

### 2.5 Wound‐healing assay

Cells were seeded in 6‐cm dishes at a density of 1 × 10^6^ cells in 5 mL medium. When cells were adherent and confluent, a wound line was made using a 200‐μL pipette tip. Images were acquired in different time by an Olympus Imaging System Microscope (magnification 40×). Experiment was performed in triplicate, and the area was measured using Image J software.

### 2.6 In vitro invasion assays

Cells were serum‐starved in medium with 0.1% FBS for 12 hours before use. Invasion assay was performed using chambers containing 8.0‐μm pore size membranes (CORING, USA) coated with Matrigel (BD Biosciences, NJ, USA). A single cell suspension (1 × 10^4^) in 0.1% bovine serum albumin (BSA) medium was seeded into the chamber. Subsequently, chambers were placed into each well of a 24‐well plate, with their bottom immersed in 10% FBS medium, and then stored at 37°C. After respective time for each group, the chamber was transferred to a crystal violet solution for 20 minutes at 37°C. The Matrigel and cells on the top side were scraped off by a wet cotton swab. Pictures were taken using an Olympus Imaging System Microscope (magnification 100×).

### 2.7 Stable transfection of prostate cancer cells with NDR1

The method of NDR1 gene silenced by Lentivirus infection. In brief, packaging vectors including pMD2.0G and psPAX (Invitrogen, CA, USA) were co‐transfected with sh‐NDR1 or control vectors into HEK293T cells using Lipofectamine 2000 (Invitrogen, CA, USA) according to the manufacturer's protocol. After 36‐hour incubation, the supernatant containing viral particles was collected, filtrated and used to infect PC3 and LNCaP cancer cells. Therefore, we got control group: PC3‐Mock and LNCaP‐Mock, NDR1 silenced group: PC3‐N3&N5 and LNCaP‐N5.Puromycin was used for selecting infected cells needed for the next experiments. To obtain overexpression protein of NDR1, pClneoMyc human NDR1 Plasmid (Addgene #37023, from Yutaka Hata) was transfected into CTC and C4‐2 cancer cell using Lipofectamine 2000 following the manufacturer's protocol. Then, transfected cells were selected using a concentration of 400‐600 μg/mL and maintained under 200‐400 μg/mL G‐418. Finally, we got control group: C4‐2‐EV and CTC‐EV, NDR1 upregulated group: CTC‐NDR1‐1&CTC‐NDR1‐2 and C4‐2‐NDR1‐1.

### 2.8 Immunohistochemistry

All the prostate cancer specimens were obtained from the Department of Urology and diagnosed by the Department of Pathology of Xijing Hospital. Tissue slides were deparaffinized in xylene baths and rehydrated in different concentration of alcohol. Subsequently, slides were boiled in sodium citrate buffer (10 mmol/L, pH 9.0) for 20 minutes and washed in PBS for antigen retrieval. Quenching of endogenous peroxidase was performed using 1.5% hydrogen peroxide, and samples were blocked in 5% goat serum for 20 minutes. Slides were incubated in NDR1 antibody overnight, washed in PBS, and incubated in HRP‐conjugated secondary antibody for 1 hour at room temperature. DAB reagent was used for chromogenic staining. Slides were dehydrated in subsequent baths in alcohol and then in xylene. Finally, slides were mounted, covered with cover slips, and scanned using Olympus Imaging System Microscope. NDR1 mouse monoclonal antibody (1:50) for immunohistochemistry was purchased from Sigma‐Aldrich (Saint Louis, USA). Immunohistochemistry evaluation was performed according to IRS proposed by Remmele and StegnerI,^31^ defined as staining intensity (SI) multiplied by the percentage of positive cells (PP). SI was scored as 0 (negative), 1 (weak), 2 (moderate), and 3 (strong). PP was defined as 0 (negative), 1 (≤10% positive cells), 2 (11%‐50% positive cells), 3 (51%‐80% positive cells), and 4 (>80% positive cells).

### 2.9 Immunofluorescence staining

Cells were seeded on a millicell slide (Millipore, Billerica, MA, USA) and incubated until cell stretched. Paraformaldehyde (4%) was used to fixate cell and then Trition‐X100 (0.3%) used to increase the membrane permeability. After blocking by goat serum, the slide was incubated in primary antibody against E‐cadherin, N‐cadherin, Snail, vimentin, β‐catenin (Cell Signaling Technology, Beverly, MA, USA) at 4° overnight. Next, slide was incubated with fluorochrome‐labeled secondary antibody (Alexa Fluor 488; life technologies, USA) at room temperature and for 30 minutes. DAPI was used to stain nucleus. Finally, observing the result of staining and getting pictures under confocal laser‐scanning microscopy (FluoView FV10i; Olympus, Japan).

### 2.10 Gene expression analysis

Total RNA of PC3 cell line and CTC was extracted and purified using Qiagen RNeasy kit (Qiagen, San Diego, CA, USA). RNA quality was evaluated by Agilent 2100 Bioanalyzer (Agilent, CA, USA). Gene expression profiling was performed using cDNA microarrays (Affymetrix GeneChip PrimeView Human Gene Expression Array) by GeneChem Co. (Shanghai, China). RAW data were analyzed, and fold changes and P‐value by were obtained affy packages. These data are available at GEO with accession number: GSE106363. Heatmap was drawn by pheatmap packages in R software.

### 2.11 Gene expression data and clinical information of patients with prostate cancer from GEO

We downloaded gene expression data and clinical information of patients with prostate cancer form GEO database (GSE16560 & GSE21034) and R script was used to extract the expression values of interest; then, a graph was draw using PRISM5.0 software (GraphPad Software, CA, USA).

### 2.12 EMT PCR array

Total RNA was extracted from PC3‐MOCK and PC3‐N5 cells using RNeasy Plus Mini Kit (Qiagen, San Diego, CA, USA) and reverse‐transcribed to cDNA by RT2 First Strand Kit (Qiagen, San Diego, CA, USA). Next, cDNA was subjected to Human EMT PCR Array (Qiagen, San Diego, CA, USA) using RT2 SYBR Green ROX qPCR Mastermix (Qiagen, San Diego, CA, USA) following the manufacturer's protocol. The fluorescence signal was detected by 7500 fast real‐time cycler (Applied Biosystems, CA, USA). Finally, data were analyzed using PCR array analysis tool online (SABioscience, CA, USA).

### 2.13 Animals and lung metastasis model

The protocol for the animal study was approved by the Ethics Committee of the Fourth Military Medical University (Xi'an, China). Male athymic BALB/c nude mice (8 weeks) were purchased from and housed at The Center of Laboratory Animal of FMMU. As for lung metastasis model, 2 × 10^6^ tumor cells in 200 μL PBS were injected into the tail vein of each mouse. Mice were sacrificed 15‐30 days after injection, and lungs were removed and dissected for counting visible metastatic lesions. For elevating numbers of metastatic lesions, 3 sections of each group were randomly chosen and metastatic lesions were counted. The count was performed 3 times for each section.

### 2.14 Statistical analysis

Student's *t* test was used to evaluate statistical significance between 2 unpaired data and log‐rank Test was used to determine the significance, if present, in the survival curve values. Ranked data were analyzed using Mann‐Whitney test. For all statistical tests, 2‐tailed *P*‐value <.05 was considered statistically significant.

## 3 RESULTS

### 3.1 Gene expression analysis of CTCs and PC3 cell line by Microarray

**Figure 1:**
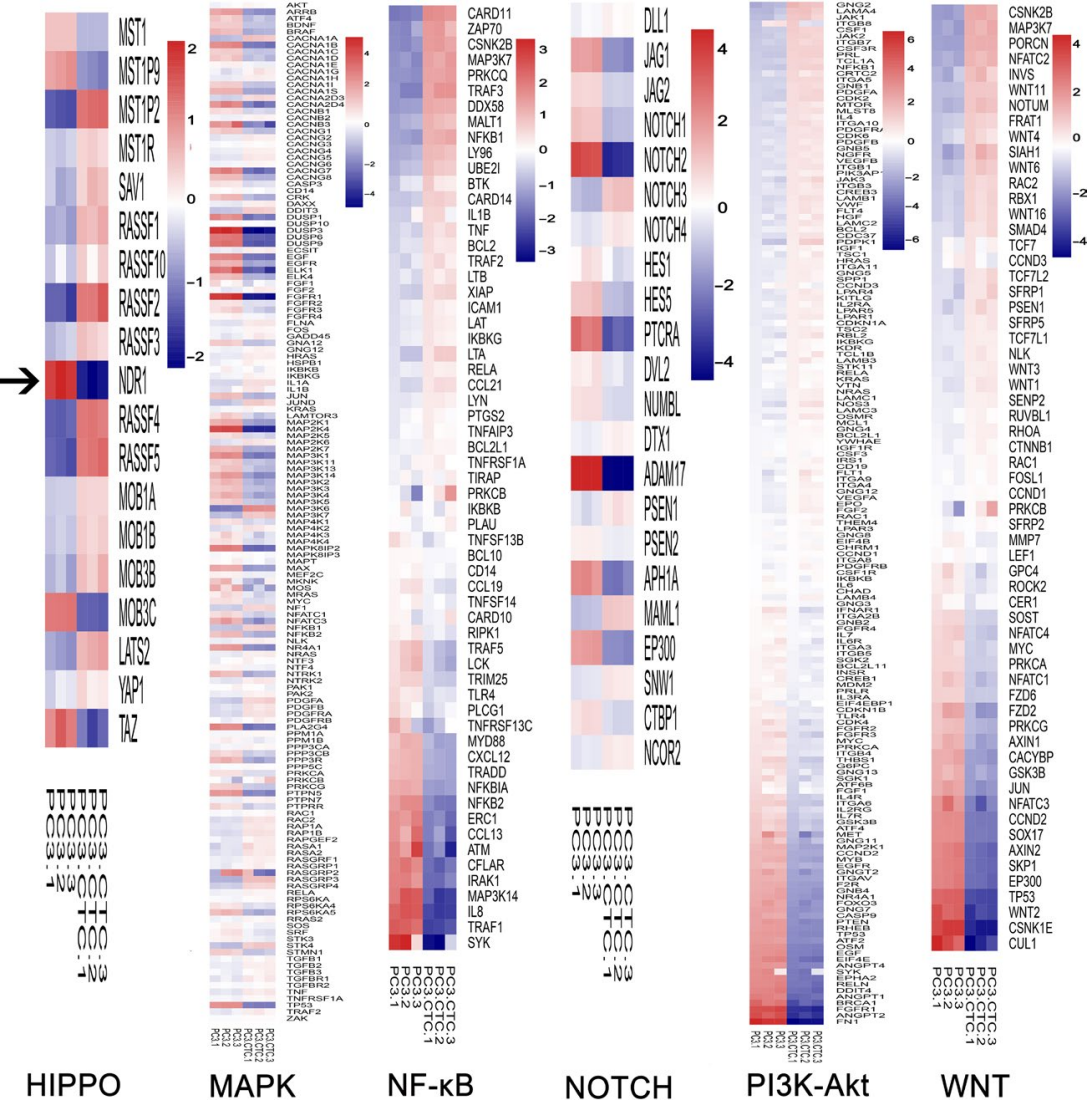
Identification of NDR1 as a potential metastasis suppressor gene. Gene expression analysis of 6 major signaling pathways involved in metastasis of PC3 cells (HIPPO, MAPK, NOTCH, WNT, PI3K‐Akt, and NF‐κB) using cDNA microarrays. NDR1, a member of HIPPO pathway, was chosen for further research (the black arrow). The figure shows different expression in 19 Hippo pathway associated genes between PC3 and CTCs cell line. NDR1 fold change in CTCs vs PC3 was −5.13, with a *P* value = .00018

To explore the pathway characterizing the higher invasiveness ability of CTCs compared with other cell types, gene expression of CTCs and its original primary cancer cells PC3 was analyzed to evaluate a potential difference. According to previous studies, we chosen and analyzed 6 major cell signaling pathways (MAPK, HIPPO, PI3K‐Akt, NOTCH, WNT and NF‐κB), which are altered during the development and progression of prostate cancer.^32^, ^33^ In particular, we put our sight on molecular in Hippo pathway due to the remarkable difference between the 2 cell types and less related study available. To demonstrate the difference in Hippo pathway between these 2 cell types, 19 major genes according to the map of Hippo pathway on the KEGG pathway database were selected and a heatmap was used to display these differences (Figure 1). As this figure shows, NDR1 mRNA was the one with the most remarkable difference in expression between CTCs and PC3. Its fold change between CTCs and PC3 was −5.13, with a *P*‐value = .0002, suggesting that NDR1 mRNA in CTCs was 5.13 times downregulated than PC3.

### 3.2 NDR1 is significantly downregulated during prostate cancer metastasis

**Figure 2:**
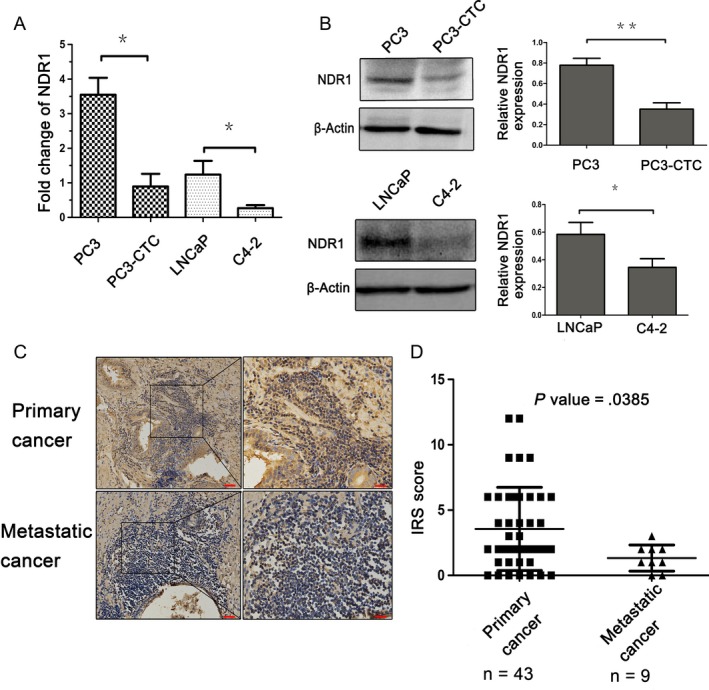
NDR1 reduced expression in prostate cancer cell and tissues metastasis. A, mRNA expression in PC3 and PC3‐CTC cells and LNCaP and C4‐2 cells. GAPDH mRNA was used as an internal control. Each experiment was repeated 3 times. *P <* *.05, **P < .01 by Student's *t* test. B, Protein expression analysis in PC3 and CTCs cells and LNCaP and C4‐2 cells. β‐actin was used as an internal control. Each experiment was repeated 3 times. *P < .05, **P < .01 by Student's *t* test. C, Analysis of NDR1 protein expression by immunohistochemistry in primary and metastatic prostate cancer. Magnification images taken at 200× (red bar = 50 μm) and 400× (red bar = 20 μm). D, NDR1 expression in samples evaluated by IRS score, Mann‐Whitney test

To verify our previous gene array findings, 2 pairs of cells were chosen and NDR1 expression in all of them was examined at mRNA and protein level. One pair of cells was represented by PC3 and CTCs generated form PC3,^29^ and the other pair was LNCaP and its derivative cell line C4‐2.^30^ Compared with 2 parental cells, the derived cells have stronger metastatic ability. Indeed, our results showed that CTCs had 3‐ to 4‐fold less NDR1 mRNA and approximately twofold less NDR1 protein than PC3, and similar results were obtained in LNCaP and C4‐2, as C4‐2 NDR1 mRNA and protein expression were respectively 3‐ and 1.5‐fold less than in LNCaP (Figure 2A,B). To further confirm our results, 81 clinical specimens were included and NDR1 was examined using immunohistochemistry. NDR1 was detected in prostate cancer tissues grouped by tumor status. Specifically, NDR1 displayed higher expression in primary prostate cancer than metastasis (Figure 2C). Next, NDR1 expression was analyzed according to the staining score IRS. NDR1 expression decreased as malignancy increased; thus, its expression was decreasing from primary cancer to metastasis (Figure 2D).

### 3.3 NDR1 suppresses prostate cancer cells metastatic potential

**Figure 3:**
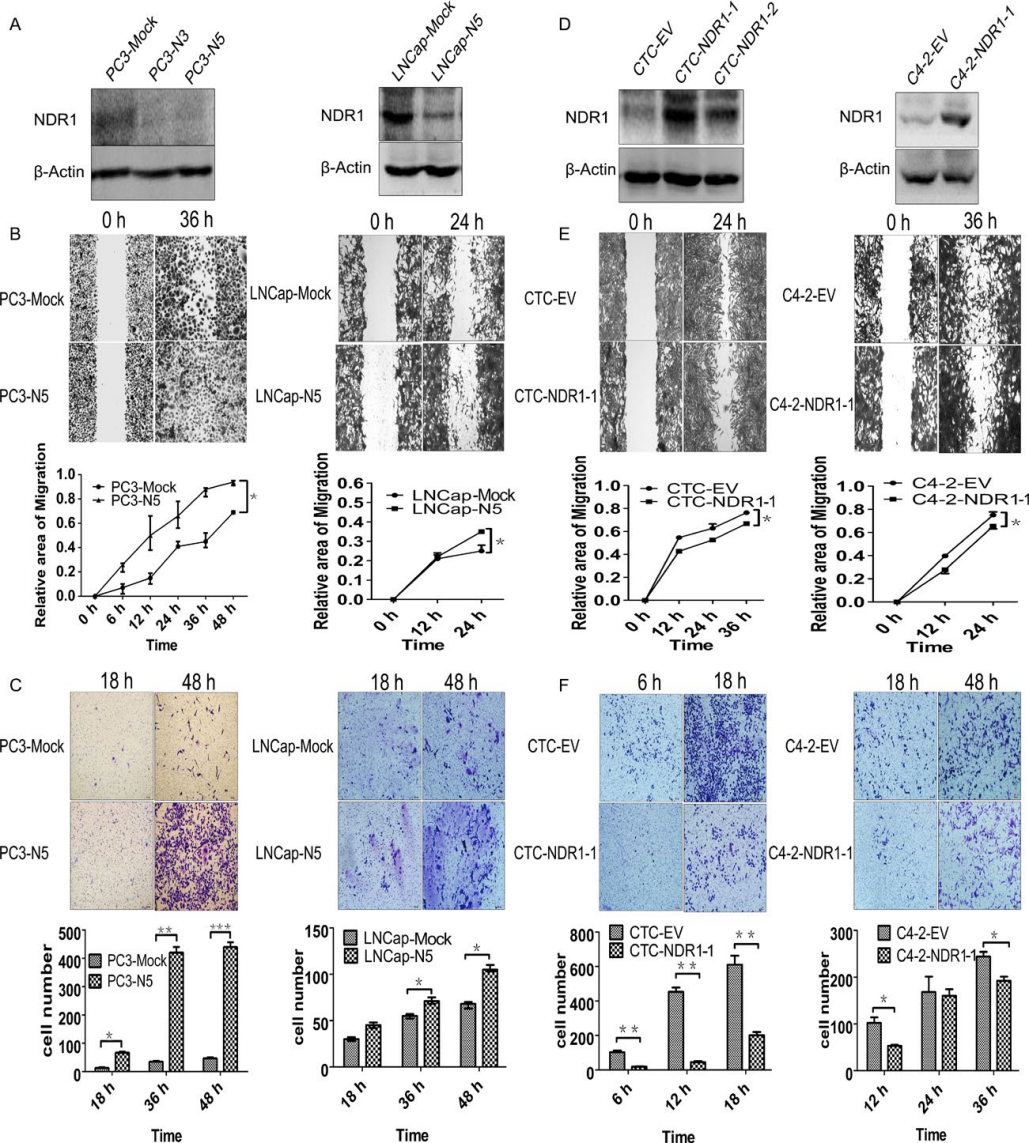
NDR1 downregulation promoted prostate cancer cell metastatic ability in vitro. A, PC3 or LNCaP cell line were transfected with shRNA against NDR1 (N3 or N5) and Mock. Western blot confirmed transfection efficiency and β‐actin was used as an internal control. B, Wound‐healing assay showing NDR1 downregulation associated to enhanced prostate cancer cell migration. Images were taken at 0, 6, 12, 24, 36, 48 h for PC3 cell (0, 12, 24 for LNCaP cell) after scratching (40× magnification). Migration area was measured by Image J software. *P < .05, **P < .01 by Student's *t* test. C, NDR1 downregulation promoted PC3 and LNCaP cell invasion. Invasive cell number was counted, respectively, at 18, 36, 48 h under microscope at 100× magnification. *P < .05, **P < .01, ***P < .001 by Student's *t* test. D, To overexpress NDR1, PC3‐CTC and C4‐2 cells were transfected with pClneoMyc human NDR1 plasmids and empty vector. Western blot was performed to examine transfection efficiency, and β‐actin was used as an internal control. E, PC3‐CTC or C4‐2 cell migration reduced by NDR1 overexpression. Images were taken at 0, 12, 24, 36 h for PC3‐CTC (0, 12, 24 for C4‐2 cell) after scratching (40× magnification). Migration area was measured by Image J software. *P < .05, **P < .01, Student's *t* test. F, NDR1 overexpression attenuated PC3‐CTC and C4‐2 cell invasion. Invasive cell number was counted at 6, 12, 18 h for PC3‐CTC (12, 24, 36 h for C4‐2) under 100× magnification. *P < .05, **P < .01, ***P < .001 by Student's *t* test

To test whether NDR1 could play a role as a metastasis suppressor in prostate cancer cell, NDR1 was knockdown in 2 cell lines (PC3 and LNCaP) (Figure 3A), which have weaken metastatic ability and expressed NDR1 at high level compared with the other 2 cell lines. Next, migration and invasion abilities were evaluated using wound‐healing assays and transwell assay, respectively. The results clearly showed that PC3‐N5 and LNCaP‐N5 cells healed the gaps faster than PC3‐Mock and LNCaP‐Mock cells (Figure 3B). In addition, same results were observed when comparing the number of cells that passed through the membrane in the transwell chamber, such as more PC3‐N5 and LNCaP‐N5 cells crossed the membrane compared with PC3 and LNCaP cells (Figure 3C). Thus, NDR1 silencing promoted PC3 and LNCaP cell migration and invasion. As a further confirmation, we performed the opposite experiment, such as NDR1 was upregulated in CTCs and C4‐2 cells, as this gene is expressed at low level in these cells and then migration and invasion abilities were examined using the same assays (Figure 3D). NDR1 upregulation conferred to these cells a weaker ability of invasion and migration than their correspondent parental cells (Figure 3E,F). Overall, these observations suggested that NDR1 acted as a metastasis suppressor in prostate cancer cells.

### 3.4 NDR1 downregulation leads to increased lung metastasis in animal models

**Figure 4:**
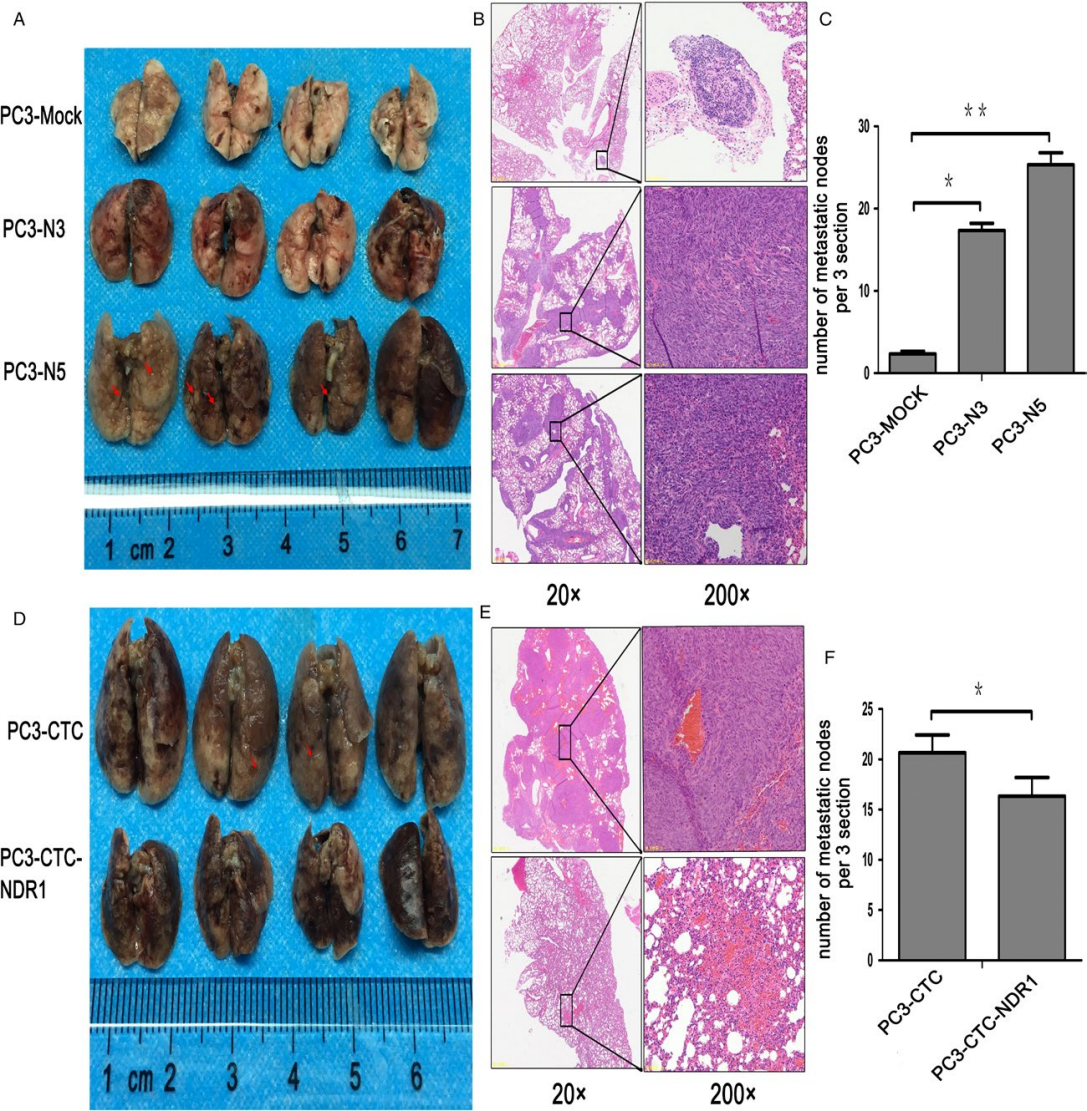
NDR1 downregulation promoted cancer cell lung metastasis in nude mice. A, Lung metastasis model was established in nude mice by injecting 2 × 10^6^ tumor cells in 200 μL PBS (PC3‐Mock, PC3‐N3 or PC3‐N5) into the lateral tail vein of each mouse (n = 5/group). Mice were sacrificed 2 weeks after injection. Images show some external metastatic nodules in the lung (red arrow). B, Representative H&E images of metastatic nodules in lung of each group in (A) (20× and 200× magnification). C, Statistical analysis results of H&E staining. Three sections of each group in (B) were randomly chosen, and metastatic nodules were counted. The count was performed 3 times for each section. *P < .05, **P < .01 by Student's *t* test. D, Lung metastasis model was established in nude mice by injecting PC3‐CTC and PC3‐CTC‐NDR1 cells, following the same protocol as described in A. E, Representative H&E images of metastatic nodules in lungs from each group in (D). F, Statistical analysis of 4E

To confirm our results in vivo, Lung Colonization Assay was performed in male athymic BALB/c nude mice to test whether NDR1 acted as a metastasis suppressor. As shown in Figure 4, the lungs of the mice injected with PC3‐N3 or PC3‐N5 cells were bigger and with more metastatic nodules than those injected with PC3‐Mock cells (Figure 4A). H&E staining confirmed the presence of more metastatic nodules in the lung of mice injected with PC3‐N3 or PC3‐N5 cells compared with those injected with PC3‐Mock cells (Figure 4B). Statistical analysis of metastatic nodules is shown in Figure 4C. Besides, the results displayed in mice injected with CTCs and mice injected with CTCs containing NDR1 overexpression also supported the same result in the opposite direction (Figure 4D‐F). Overall, the above observations suggested that NDR1 acted as a metastasis suppressor in animal models.

### 3.5 NDR1 silence contribute to activation of EMT pathway

**Figure 5:**
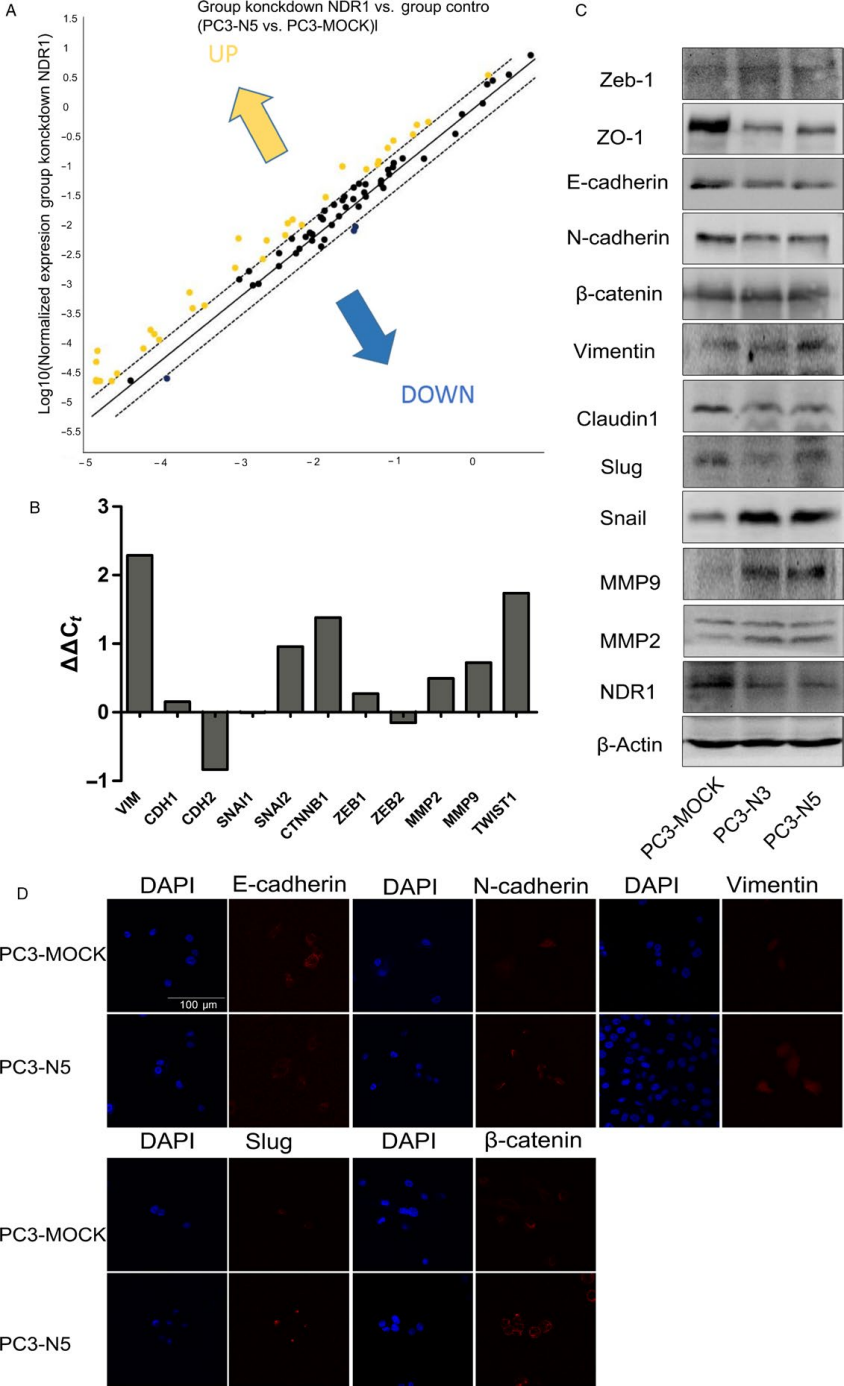
NDR1 silence contributes to activation of epithelial‐mesenchymal transition. A, Total RNA from PC3‐Mock and PC3‐N5 cells were characterized using Human EMT PCR Array. The figure shows scatter plot of different expression changes of 84 genes before and after NDR1 downregulation. B, Expression changes of 11 epithelial‐mesenchymal transition‐related genes after NDR1 downregulation. C, Western blot of some of the epithelial‐mesenchymal transition related genes after NDR1 downregulation. Marker of epithelial like ZO‐1 and E‐cadherin decrease and mesenchymal marker (snail, vimentin, MMP2, and MMP9) increase, suggesting epithelial‐mesenchymal transition activation with decreased NDR1. β‐actin was used as an endogenous control. D, Immunoflorescence staining show some EMT markers changes between PC3‐Mock (control group) and PC3‐N5 (NDR1 silence group) cells, with DAPI to visualize the nucleus. Scale bar = 100 μm

To explore NDR1 mechanism on tumor metastasis, we profiled changes in EMT associated genes using RT2 ProfilerTM Human EMT PCR Array that contains 84‐related genes (Dataset S1). The results showed that most of the EMT‐related genes were upregulated (37 of 41, while the remaining 43 genes included 12 controls and 31 genes without statistical significance) when NDR1 was downregulated, while the expression of only 3 genes was reduced (Figure 5A and Dataset S2 and S3). This result potentially suggested that EMT process was activated by NDR1 silencing. As a further confirmation, we chose 11 core genes, known as EMT markers, according to a review.^34^ They included CDH1 (also known as E‐cadherin, usually considered as a EMT suppressor) and other usually upregulated members during EMT process, such as CDH2 (also known as N‐cadherin), Snail, Slug, ZEB2, ZEB1, CTNNB1, Vimentin, Twist, MMP2&9. Results showed that most of the mesenchymal markers were upregulated supporting our previous findings (Figure 5B). However, not all results obtained were according our expectations, as E‐cadherin was increased and CDH2 and ZEB2 were decreased in mRNA level, while we were expecting opposite results. However, the level of protein expression in E‐cadherin was in accordance with our previous expectations from the Western blot analysis. Besides, ZEB1, Vimentin, MMP2, and MMP9 protein expression measured by Western blot was in accordance with the correspondent mRNAs expression (Figure 5C). Thus, the above‐mentioned protein may be our potential research objects in the future. Moreover, the immunofluorescence staining of some EMT markers changes was consistent with the Western blot results (Figure 5D). Collectively, an enhanced EMT process was observed when NDR1 was silenced. Based on the above results, we speculate that NDR1 might act as a metastasis suppressor by inhibiting EMT process.

### 3.6 NDR1 decrease is associated to poor prognosis in prostate cancer patients

**Figure 6:**
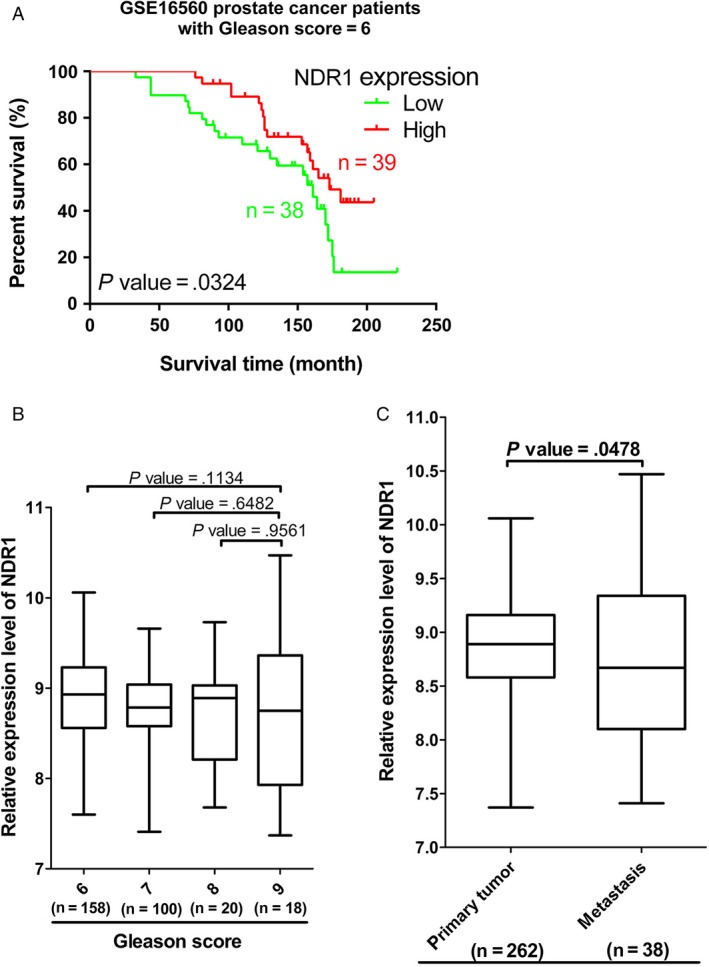
NDR1 low expression was related to poor clinical outcome. A, Kaplan‐Meier analysis depicted a correlation between NDR1 expression and overall survive time. The analysis was based patients (Gleason score = 6 and removed samples which Fusion is Not Applicable) in GSE16560. B, NDR1 expression analysis in GSE21034 grouped by Gleason score. C, NDR1 expression analysis in GSE21034 grouped based on tumor status

To investigate NDR1clinical significance, we obtained follow survive time (in month) of patients with prostate cancer from GEO (GSE16560) and analyzed whether there was correlation between NDR1 expression and overall survive time. We found that the survival time was longer when NDR1 was highly expressed compared with NDR1 low expression (Figure 6A). These results suggest that decreased NDR1 expression might led to poorer patients’ prognosis. Besides, data of prostate cancer patients cohort downloaded from GEO (GSE 21034) were further analyzed. As we found above, NDR1 expression was lower in metastatic prostate cancer than in primary prostate cancer, with both comparison statistically different. However, expression of NDR1 had no statistically difference within each group divided by Gleason score (Figure 6B,C).

## 4 DISCUSSION

Metastatic behavior of tumor is complex progression, which usually is accompanied by disorder of some particular genes and pathway.^35^ Here, we reported a novel prostate cancer metastasis suppressor, NDR1, by analyzing differences in gene expression between CTCs and its parental cell line, PC3. In our study, we firstly investigated NDR1 expression in different cell line and clinical samples. The expression of NDR1 was lower in metastatic cell with stronger invasive ability than primary cancer cell, and the same results were found in samples of patients with prostate cancer. Then, we examine the capability of migration and invasion in cell with either downregulated NDR1 or upregulated. It suggests that NDR1 acts as a metastasis inhibitor in prostate cancer both in vivo or in vitro. We further found that NDR1 decrease was positively associated with poor prognosis in prostate cancer patients. Besides, an activated EMT process was observed with depleted NDR1, which meant NDR1 might inhibit tumor metastasis by depressing EMT.

NDR1 is usually reported and plays an important role in various biological processes. As regard cancer, few studies are available and most of them reported NDR1 as a tumor suppressor. H. Cornils reported that NDR1 ablation in mice is associated with the development of T‐cell lymphoma.^36^ Results of microarray in many human cancers also displayed that NDR1 was decreased compared with their corresponding normal tissues, such as gastric cancer,^37^ skin cancer,^38^ acute lymphoblastic leukemia,^39^ and prostate cancer.^13^, ^14^, ^15^, ^16^ Conversely, opposite result was found in breast cancer.^9^ Collectively, notion that NDR1 acts as tumor suppressor protein has been strengthened in most though few reports, so as our result.

We explored the potential molecular mechanism underlined prostate cancer process of metastasis and revealed a connection between NDR1 decrease and EMT increase, although not all the parameters we considered to demonstrate the relationship between NDR1 and EMT were significant or coherent. As NDR1 is a member of NDR kinase subgroup in Hippo pathway and has 3 related kinases NDR2, LATS1, and LATS2, the 4 kinases sometimes display overlapping functions.^40^ Mammalian Hippo pathway is usually identified as putative tumor suppression associated pathway^19^ and its function is achieved mainly through YAP/TAZ oncoprotein.^41^ Similarly, upregulation and stabilization of YAP/TAZ in tumor mainly correspond to an inactivation of the Hippo pathway.^42^, ^43^ Recent studies also showed NDR1/2 kinase can phosphorylate YAP1 on S127 and negatively regulated YAP1 activity^44^ and same results were also found in Lats kinases.^45^ Consequently, YAP and TAZ overexpression led to EMT promotion^46^, ^47^ that can be inhibited by Hippo pathway. Taken together, these reports support the evidence that decreased NDR1 lead to EMT promotion. However, our data demonstrated this phenomenon in prostate cancer metastasis, revealing the invasion of the lungs. Indeed, our results revealed NDR1 as antimetastasis candidate and its expression and function in vitro and in vivo was evaluated, which was beneficial for us to understand the mechanism of prostate cancer metastasis. Besides, NDR1 might be a novel marker for predicting clinical outcome of prostate cancer patients. However, further investigation is needed to understand our unclear results associated with CDH2 and ZEB2.

Our exploration is insufficient in special molecular mechanism aspect, directed and detailed interactions between NDR1 and EMT‐related molecular are not revealed. Thus, further studies are needed to understand how NDR1 affects on procession of EMT.

In summary, we analyzed the difference in gene expression between CTC and PC3 cells and demonstrated that NDR1 expression decreased in prostate cancer cell that have strong metastatic ability, especially those with metastatic patients, showing correlation between decreased NDR1 and poor prognosis. The demonstration that NDR1 inhibited prostate cancer migration and invasion was performed in vitro and in vivo. Finally, our data also indicated that NDR1 inactivation resulted in an activation of EMT, leading to metastasis. Therefore, NDR1 might be considered as a new marker during cancer progression and might be beneficial in the treatment of prostate cancer patients.

## CONFLICT OF INTEREST

All authors state that there are no conflicts of interest to declare.
