# Supplementary material for: Downregulation of NDR1 contributes to metastasis of prostate cancer cells via activating epithelial‐mesenchymal transition
Source: Cancer Med. 2018 May 7;7(7):3200–12. doi: 10.1002/cam4.1532 (PMC6051198; doi:10.1002/cam4.1532)
Supplement: Supplementary file 1 [file CAM4-7-3200-s001.docx]

**Position UniGene GenBank Symbol Description**

A01 Hs.502756 NM_024060 AHNAK AHNAK nucleoprotein

A02 Hs.525622 NM_005163 AKT1 V-akt murine thymoma viral oncogene homolog 1

A03 Hs.1274 NM_006129 BMP1 Bone morphogenetic protein 1

A04 Hs.73853 NM_001200 BMP2 Bone morphogenetic protein 2

A05 Hs.473163 NM_001719 BMP7 Bone morphogenetic protein 7

A06 Hs.490203 NM_004342 CALD1 Caldesmon 1

A07 Hs.197922 NM_018584 CAMK2N1 Calcium/calmodulin-dependent protein kinase II inhibitor 1

A08 Hs.212332 NM_001233 CAV2 Caveolin 2

A09 Hs.461086 NM_004360 CDH1 Cadherin 1, type 1, E-cadherin (epithelial)

A10 Hs.464829 NM_001792 CDH2 Cadherin 2, type 1, N-cadherin (neuronal)

A11 Hs.489142 NM_000089 COL1A2 Collagen, type I, alpha 2

A12 Hs.443625 NM_000090 COL3A1 Collagen, type III, alpha 1

B01 Hs.445827 NM_000393 COL5A2 Collagen, type V, alpha 2

B02 Hs.476018 NM_001904 CTNNB1 Catenin (cadherin-associated protein), beta 1, 88kDa

B03 Hs.95612 NM_004949 DSC2 Desmocollin 2

B04 Hs.519873 NM_004415 DSP Desmoplakin

B05 Hs.488293 NM_005228 EGFR Epidermal growth factor receptor

B06 Hs.118681 NM_001982 ERBB3 V-erb-b2 erythroblastic leukemia viral oncogene homolog 3 (avian)

B07 Hs.208124 NM_000125 ESR1 Estrogen receptor 1

B08 Hs.517293 NM_016946 F11R F11 receptor

B09 Hs.1690 NM_005130 FGFBP1 Fibroblast growth factor binding protein 1

B10 Hs.203717 NM_002026 FN1 Fibronectin 1

B11 Hs.436448 NM_005251 FOXC2 Forkhead box C2 (MFH-1, mesenchyme forkhead 1)

B12 Hs.173859 NM_003507 FZD7 Frizzled family receptor 7

C01 Hs.83381 NM_004126 GNG11 Guanine nucleotide binding protein (G protein), gamma 11

C02 Hs.440438 NM_173849 GSC Goosecoid homeobox

C03 Hs.445733 NM_002093 GSK3B Glycogen synthase kinase 3 beta

C04 Hs.462998 NM_001552 IGFBP4 Insulin-like growth factor binding protein 4

C05 Hs.81134 NM_000577 IL1RN Interleukin 1 receptor antagonist

C06 Hs.5158 NM_004517 ILK Integrin-linked kinase

C07 Hs.505654 NM_002205 ITGA5 Integrin, alpha 5 (fibronectin receptor, alpha polypeptide)

C08 Hs.436873 NM_002210 ITGAV Integrin, alpha V (vitronectin receptor, alpha polypeptide, antigen CD51)

C09 Hs.643813 NM_002211 ITGB1 Integrin, beta 1 (fibronectin receptor, beta polypeptide, antigen CD29 includes MDF2, MSK12)

C10 Hs.728907 NM_000214 JAG1 Jagged 1

C11 Hs.654380 NM_000526 KRT14 Keratin 14

C12 Hs.654568 NM_002276 KRT19 Keratin 19

D01 Hs.411501 NM_005556 KRT7 Keratin 7

D02 Hs.335079 NM_005909 MAP1B Microtubule-associated protein 1B

D03 Hs.513617 NM_004530 MMP2 Matrix metallopeptidase 2 (gelatinase A, 72kDa gelatinase, 72kDa type IV collagenase)

D04 Hs.375129 NM_002422 MMP3 Matrix metallopeptidase 3 (stromelysin 1, progelatinase)

D05 Hs.297413 NM_004994 MMP9 Matrix metallopeptidase 9 (gelatinase B, 92kDa gelatinase, 92kDa type IV collagenase)

D06 Hs.87752 NM_002444 MSN Moesin

D07 Hs.517973 NM_002447 MST1R Macrophage stimulating 1 receptor (c-met-related tyrosine kinase)

D08 Hs.370414 NM_018055 NODAL Nodal homolog (mouse)

D09 Hs.495473 NM_017617 NOTCH1 Notch 1

D10 Hs.533657 NM_015901 NUDT13 Nudix (nucleoside diphosphate linked moiety X)-type motif 13

D11 Hs.592605 NM_002538 OCLN Occludin

D12 Hs.509067 NM_002609 PDGFRB Platelet-derived growth factor receptor, beta polypeptide

E01 Hs.170473 NM_016445 PLEK2 Pleckstrin 2

E02 Hs.570455 NM_015704 PPPDE2 PPPDE peptidase domain containing 2

E03 Hs.395482 NM_005607 PTK2 PTK2 protein tyrosine kinase 2

E04 Hs.227777 NM_003463 PTP4A1 Protein tyrosine phosphatase type IVA, member 1

E05 Hs.413812 NM_006908 RAC1 Ras-related C3 botulinum toxin substrate 1 (rho family, small GTP binding protein Rac1)

E06 Hs.78944 NM_002923 RGS2 Regulator of G-protein signaling 2, 24kDa

E07 Hs.414795 NM_000602 SERPINE1 Serpin peptidase inhibitor, clade E (nexin, plasminogen activator inhibitor type 1), member 1

E08 Hs.708127 NM_003616 SIP1 Survival of motor neuron protein interacting protein 1

E09 Hs.12253 NM_005901 SMAD2 SMAD family member 2

E10 Hs.48029 NM_005985 SNAI1 Snail homolog 1 (Drosophila)

E11 Hs.360174 NM_003068 SNAI2 Snail homolog 2 (Drosophila)

E12 Hs.253790 NM_178310 SNAI3 Snail homolog 3 (Drosophila)

F01 Hs.376984 NM_006941 SOX10 SRY (sex determining region Y)-box 10

F02 Hs.111779 NM_003118 SPARC Secreted protein, acidic, cysteine-rich (osteonectin)

F03 Hs.313 NM_000582 SPP1 Secreted phosphoprotein 1

F04 Hs.463059 NM_003150 STAT3 Signal transducer and activator of transcription 3 (acute-phase response factor)

F05 Hs.61635 NM_012449 STEAP1 Six transmembrane epithelial antigen of the prostate 1

F06 Hs.371282 NM_003200 TCF3 Transcription factor 3 (E2A immunoglobulin enhancer binding factors E12/E47)

F07 Hs.644653 NM_003199 TCF4 Transcription factor 4

F08 Hs.438231 NM_006528 TFPI2 Tissue factor pathway inhibitor 2

F09 Hs.645227 NM_000660 TGFB1 Transforming growth factor, beta 1

F10 Hs.133379 NM_003238 TGFB2 Transforming growth factor, beta 2

F11 Hs.592317 NM_003239 TGFB3 Transforming growth factor, beta 3

F12 Hs.522632 NM_003254 TIMP1 TIMP metallopeptidase inhibitor 1

G01 Hs.598100 NM_003692 TMEFF1 Transmembrane protein with EGF-like and two follistatin-like domains 1

G02 Hs.118552 NM_178031 TMEM132A Transmembrane protein 132A

G03 Hs.364544 NM_014399 TSPAN13 Tetraspanin 13

G04 Hs.66744 NM_000474 TWIST1 Twist homolog 1 (Drosophila)

G05 Hs.643801 NM_004385 VCAN Versican

G06 Hs.642813 NM_003380 VIM Vimentin

G07 Hs.459790 NM_033305 VPS13A Vacuolar protein sorting 13 homolog A (S. cerevisiae)

G08 Hs.108219 NM_004626 WNT11 Wingless-type MMTV integration site family, member 11

G09 Hs.696364 NM_003392 WNT5A Wingless-type MMTV integration site family, member 5A

G10 Hs.306051 NM_032642 WNT5B Wingless-type MMTV integration site family, member 5B

G11 Hs.124503 NM_030751 ZEB1 Zinc finger E-box binding homeobox 1

G12 Hs.34871 NM_014795 ZEB2 Zinc finger E-box binding homeobox 2

H01 Hs.520640 NM_001101 ACTB Actin, beta

H02 Hs.534255 NM_004048 B2M Beta-2-microglobulin

H03 Hs.592355 NM_002046 GAPDH Glyceraldehyde-3-phosphate dehydrogenase

H04 Hs.412707 NM_000194 HPRT1 Hypoxanthine phosphoribosyltransferase 1

H05 Hs.546285 NM_001002 RPLP0 Ribosomal protein, large, P0

H06 N/A SA_00105 HGDC Human Genomic DNA Contamination

H07 N/A SA_00104 RTC Reverse Transcription Control

H08 N/A SA_00104 RTC Reverse Transcription Control

H09 N/A SA_00104 RTC Reverse Transcription Control

H10 N/A SA_00103 PPC Positive PCR Control

H11 N/A SA_00103 PPC Positive PCR Control

H12 N/A SA_00103 PPC Positive PCR Control
