# Supplementary material for: Downregulation of NDR1 contributes to metastasis of prostate cancer cells via activating epithelial‐mesenchymal transition
Source: Cancer Med. 2018 May 7;7(7):3200–12. doi: 10.1002/cam4.1532 (PMC6051198; doi:10.1002/cam4.1532)
Supplement: Supplementary file 2 [file CAM4-7-3200-s002.docx]

| Block Type | 96fast |  |  |  |  |  |  |
| --- | --- | --- | --- | --- | --- | --- | --- |
| Chemistry | SYBR_GREEN |  |  |  |  |  |  |
| Experiment File Name | D:\YJT QPCR\PCRARRAY-pc3-n520170807.eds |  |  |  |  |  |  |
| Experiment Run End Time | 2017-08-07 20:12:00 PM CST |  |  |  |  |  |  |
| Instrument Type | sds7500fast |  |  |  |  |  |  |
| Passive Reference | ROX |  |  |  |  |  |  |
| Baseline Start | 3 |  |  |  |  |  |  |
| Baseline End | 15 |  |  |  |  |  |  |
| Ct Threshold | 0.2 |  |  |  |  |  |  |
| HIGHSD | Y |  |  |  |  |  |  |
| NOAMP | N |  |  |  |  |  |  |
| MTP | N |  |  |  |  |  |  |
| Reporter | SYBR |  |  |  |  |  |  |
| Quencher | None |  |  |  |  |  |  |
| **Well** | **Sample Name** | **Cт** | **Cт Mean** | **Cт SD** | **Tm1** | **Tm2** | **Tm3** |
| A1 | pc3-mock | 24.37363 | 25.5561 | 4.698263 | 85.13202 |  |  |
| A2 | pc3-mock | 23.45958 | 25.5561 | 4.698263 | 80.06207 |  |  |
| A3 | pc3-mock | 26.54607 | 25.5561 | 4.698263 | 84.15074 |  |  |
| A4 | pc3-mock | 28.8708 | 25.5561 | 4.698263 | 87.74877 |  |  |
| A5 | pc3-mock | 34.33747 | 25.5561 | 4.698263 | 81.86108 |  |  |
| A6 | pc3-mock | 24.39592 | 25.5561 | 4.698263 | 79.40788 |  |  |
| A7 | pc3-mock | 21.80842 | 25.5561 | 4.698263 | 75.48276 |  |  |
| A8 | pc3-mock | 24.54766 | 25.5561 | 4.698263 | 85.13202 |  |  |
| A9 | pc3-mock | 21.19419 | 25.5561 | 4.698263 | 81.69754 |  |  |
| A10 | pc3-mock | 24.3992 | 25.5561 | 4.698263 | 83.16946 |  |  |
| A11 | pc3-mock | 31.95478 | 25.5561 | 4.698263 | 82.02464 |  |  |
| A12 | pc3-mock | 30.33955 | 25.5561 | 4.698263 | 78.59015 |  |  |
| B1 | pc3-mock | 25.24519 | 25.5561 | 4.698263 | 76.95468 |  |  |
| B2 | pc3-mock | 23.92913 | 25.5561 | 4.698263 | 83.16946 |  |  |
| B3 | pc3-mock | 26.39789 | 25.5561 | 4.698263 | 77.93596 |  |  |
| B4 | pc3-mock | 22.7346 | 25.5561 | 4.698263 | 80.06207 |  |  |
| B5 | pc3-mock | 22.63929 | 25.5561 | 4.698263 | 82.18818 |  |  |
| B6 | pc3-mock | 25.98211 | 25.5561 | 4.698263 | 80.71626 |  |  |
| B7 | pc3-mock | 34.94494 | 25.5561 | 4.698263 | 85.29557 |  |  |
| B8 | pc3-mock | 23.37006 | 25.5561 | 4.698263 | 81.04335 |  |  |
| B9 | pc3-mock | 25.31145 | 25.5561 | 4.698263 | 82.35173 |  |  |
| B10 | pc3-mock | 20.70685 | 25.5561 | 4.698263 | 81.04335 |  |  |
| B11 | pc3-mock | 32.65983 | 25.5561 | 4.698263 | 85.45911 |  |  |
| B12 | pc3-mock | 26.57769 | 25.5561 | 4.698263 | 83.16946 |  |  |
| C1 | pc3-mock | 29.02109 | 25.5561 | 4.698263 | 78.26305 |  |  |
| C2 | pc3-mock | 36.34435 | 25.5561 | 4.698263 | 85.94975 |  |  |
| C3 | pc3-mock | 22.74534 | 25.5561 | 4.698263 | 80.55271 |  |  |
| C4 | pc3-mock | 25.80187 | 25.5561 | 4.698263 | 85.78621 |  |  |
| C5 | pc3-mock | 23.84136 | 25.5561 | 4.698263 | 81.2069 |  |  |
| C6 | pc3-mock | 24.25836 | 25.5561 | 4.698263 | 80.87981 |  |  |
| C7 | pc3-mock | 25.69697 | 25.5561 | 4.698263 | 79.89853 |  |  |
| C8 | pc3-mock | 24.31098 | 25.5561 | 4.698263 | 78.59015 |  |  |
| C9 | pc3-mock | 17.92963 | 25.5561 | 4.698263 | 80.22562 |  |  |
| C10 | pc3-mock | 22.88108 | 25.5561 | 4.698263 | 75.15567 |  |  |
| C11 | pc3-mock | 24.86154 | 25.5561 | 4.698263 | 84.15074 |  |  |
| C12 | pc3-mock | 18.16882 | 25.5561 | 4.698263 | 84.80493 |  |  |
| D1 | pc3-mock | 19.24858 | 25.5561 | 4.698263 | 85.62267 |  |  |
| D2 | pc3-mock | 26.86199 | 25.5561 | 4.698263 | 78.59015 |  |  |
| D3 | pc3-mock | 34.1016 | 25.5561 | 4.698263 | 77.93596 |  |  |
| D4 | pc3-mock | 35.87049 | 25.5561 | 4.698263 | 78.59015 |  |  |
| D5 | pc3-mock | 28.42244 | 25.5561 | 4.698263 | 78.59015 |  |  |
| D6 | pc3-mock | 22.22326 | 25.5561 | 4.698263 | 83.16946 |  |  |
| D7 | pc3-mock | 27.12931 | 25.5561 | 4.698263 | 83.82365 |  |  |
| D8 | pc3-mock | 32.2882 | 25.5561 | 4.698263 | 81.2069 |  |  |
| D9 | pc3-mock | 27.82072 | 25.5561 | 4.698263 | 85.94975 |  |  |
| D10 | pc3-mock | 26.75348 | 25.5561 | 4.698263 | 82.18818 |  |  |
| D11 | pc3-mock | 21.36191 | 25.5561 | 4.698263 | 82.02464 |  |  |
| D12 | pc3-mock | 35.71375 | 25.5561 | 4.698263 | 86.60394 | 65.50641 |  |
| E1 | pc3-mock | 23.39164 | 25.5561 | 4.698263 | 75.64631 |  |  |
| E2 | pc3-mock | 23.4069 | 25.5561 | 4.698263 | 78.26305 |  |  |
| E3 | pc3-mock | 23.6846 | 25.5561 | 4.698263 | 80.87981 |  |  |
| E4 | pc3-mock | 22.43614 | 25.5561 | 4.698263 | 75.80985 |  |  |
| E5 | pc3-mock | 20.88736 | 25.5561 | 4.698263 | 77.28178 |  |  |
| E6 | pc3-mock | 25.70765 | 25.5561 | 4.698263 | 78.26305 |  |  |
| E7 | pc3-mock | 34.83538 | 25.5561 | 4.698263 | 82.67882 |  |  |
| E8 | pc3-mock | 23.91199 | 25.5561 | 4.698263 | 79.73498 |  |  |
| E9 | pc3-mock | 22.19241 | 25.5561 | 4.698263 | 79.73498 |  |  |
| E10 | pc3-mock | 32.49963 | 25.5561 | 4.698263 | 85.45911 |  |  |
| E11 | pc3-mock | 25.32251 | 25.5561 | 4.698263 | 81.37045 |  |  |
| E12 | pc3-mock | 30.98498 | 25.5561 | 4.698263 | 84.64138 |  |  |
| F1 | pc3-mock | 36.77205 | 25.5561 | 4.698263 | 83.82365 |  |  |
| F2 | pc3-mock | 33.52219 | 25.5561 | 4.698263 | 83.33301 | 73.84729 |  |
| F3 | pc3-mock | Undetermined | 25.5561 | 4.698263 | 75.64631 |  |  |
| F4 | pc3-mock | Undetermined | 25.5561 | 4.698263 | 62.23547 |  |  |
| F5 | pc3-mock | 23.9049 | 25.5561 | 4.698263 | 79.40788 |  |  |
| F6 | pc3-mock | 23.70267 | 25.5561 | 4.698263 | 74.17439 |  |  |
| F7 | pc3-mock | 26.14894 | 25.5561 | 4.698263 | 81.86108 |  |  |
| F8 | pc3-mock | 22.20816 | 25.5561 | 4.698263 | 78.75369 |  |  |
| F9 | pc3-mock | 22.84086 | 25.5561 | 4.698263 | 82.67882 |  |  |
| F10 | pc3-mock | 18.12521 | 25.5561 | 4.698263 | 77.93596 |  |  |
| F11 | pc3-mock | 25.12376 | 25.5561 | 4.698263 | 81.86108 |  |  |
| F12 | pc3-mock | 22.45205 | 25.5561 | 4.698263 | 84.47784 |  |  |
| G1 | pc3-mock | 28.01141 | 25.5561 | 4.698263 | 77.93596 |  |  |
| G2 | pc3-mock | 26.2541 | 25.5561 | 4.698263 | 83.9872 |  |  |
| G3 | pc3-mock | 22.39021 | 25.5561 | 4.698263 | 78.26305 |  |  |
| G4 | pc3-mock | 27.13612 | 25.5561 | 4.698263 | 86.76749 |  |  |
| G5 | pc3-mock | 28.2548 | 25.5561 | 4.698263 | 76.79114 |  |  |
| G6 | pc3-mock | 25.17834 | 25.5561 | 4.698263 | 78.59015 |  |  |
| G7 | pc3-mock | 25.17089 | 25.5561 | 4.698263 | 77.44532 |  |  |
| G8 | pc3-mock | 32.9697 | 25.5561 | 4.698263 | 87.42168 | 82.51527 |  |
| G9 | pc3-mock | 24.76167 | 25.5561 | 4.698263 | 86.76749 |  |  |
| G10 | pc3-mock | 28.83998 | 25.5561 | 4.698263 | 85.94975 |  |  |
| G11 | pc3-mock | 30.85028 | 25.5561 | 4.698263 | 77.44532 |  |  |
| G12 | pc3-mock | 27.66348 | 25.5561 | 4.698263 | 79.73498 |  |  |
| H1 | pc3-mock | 17.23514 | 25.5561 | 4.698263 | 84.15074 |  |  |
| H2 | pc3-mock | 19.53799 | 25.5561 | 4.698263 | 78.09951 |  |  |
| H3 | pc3-mock | 18.35908 | 25.5561 | 4.698263 | 83.9872 |  |  |
| H4 | pc3-mock | 22.74147 | 25.5561 | 4.698263 | 76.30049 |  |  |
| H5 | pc3-mock | 16.29596 | 25.5561 | 4.698263 | 80.38917 |  |  |
| H6 | pc3-mock | Undetermined | 25.5561 | 4.698263 | 71.88474 |  |  |
| H7 | pc3-mock | 22.78825 | 25.5561 | 4.698263 | 81.534 |  |  |
| H8 | pc3-mock | 21.83657 | 25.5561 | 4.698263 | 82.02464 |  |  |
| H9 | pc3-mock | 20.79337 | 25.5561 | 4.698263 | 82.35173 |  |  |
| H10 | pc3-mock | 20.7442 | 25.5561 | 4.698263 | 81.69754 |  |  |
| H11 | pc3-mock | 21.3738 | 25.5561 | 4.698263 | 81.69754 |  |  |
| H12 | pc3-mock | 23.08632 | 25.5561 | 4.698263 | 81.86108 |  |  |
