# Supplementary material for: Downregulation of NDR1 contributes to metastasis of prostate cancer cells via activating epithelial‐mesenchymal transition
Source: Cancer Med. 2018 May 7;7(7):3200–12. doi: 10.1002/cam4.1532 (PMC6051198; doi:10.1002/cam4.1532)
Supplement: Supplementary file 3 [file CAM4-7-3200-s003.docx]

| Block Type | 96fast |  |  |  |  |  |  |
| --- | --- | --- | --- | --- | --- | --- | --- |
| Chemistry | SYBR_GREEN |  |  |  |  |  |  |
| Experiment File Name | D:\YJT QPCR\PCRARRAY-pc3-n520170807.eds |  |  |  |  |  |  |
| Experiment Run End Time | 2017-08-07 20:12:00 PM CST |  |  |  |  |  |  |
| Instrument Type | sds7500fast |  |  |  |  |  |  |
| Passive Reference | ROX |  |  |  |  |  |  |
| Baseline Start | 3 |  |  |  |  |  |  |
| Baseline End | 15 |  |  |  |  |  |  |
| Ct Threshold | 0.2 |  |  |  |  |  |  |
| HIGHSD | Y |  |  |  |  |  |  |
| NOAMP | N |  |  |  |  |  |  |
| MTP | N |  |  |  |  |  |  |
| Reporter | SYBR |  |  |  |  |  |  |
| Quencher | None |  |  |  |  |  |  |
| **Well** | **Sample Name** | **Cт** | **Cт Mean** | **Cт SD** | **Tm1** | **Tm2** | **Tm3** |
| A1 | pc3-n5 | 25.425587 | 26.4516 | 4.513439 | 85.28392 |  |  |
| A2 | pc3-n5 | 24.952662 | 26.4516 | 4.513439 | 80.18995 |  |  |
| A3 | pc3-n5 | 26.403551 | 26.4516 | 4.513439 | 84.95528 |  |  |
| A4 | pc3-n5 | 27.399565 | 26.4516 | 4.513439 | 88.73467 |  |  |
| A5 | pc3-n5 | 35.550552 | 26.4516 | 4.513439 | 82.1618 |  |  |
| A6 | pc3-n5 | 25.369471 | 26.4516 | 4.513439 | 79.53266 |  |  |
| A7 | pc3-n5 | 23.139151 | 26.4516 | 4.513439 | 75.75327 |  |  |
| A8 | pc3-n5 | 26.214947 | 26.4516 | 4.513439 | 85.28392 |  |  |
| A9 | pc3-n5 | 21.350016 | 26.4516 | 4.513439 | 81.99748 |  |  |
| A10 | pc3-n5 | 23.565527 | 26.4516 | 4.513439 | 83.47638 |  |  |
| A11 | pc3-n5 | 34.856644 | 26.4516 | 4.513439 | 82.65477 |  |  |
| A12 | pc3-n5 | 30.972424 | 26.4516 | 4.513439 | 78.54674 |  |  |
| B1 | pc3-n5 | 26.383371 | 26.4516 | 4.513439 | 77.06784 |  |  |
| B2 | pc3-n5 | 25.312235 | 26.4516 | 4.513439 | 83.14774 |  |  |
| B3 | pc3-n5 | 28.189356 | 26.4516 | 4.513439 | 77.88945 |  |  |
| B4 | pc3-n5 | 24.321533 | 26.4516 | 4.513439 | 80.02563 |  |  |
| B5 | pc3-n5 | 24.712923 | 26.4516 | 4.513439 | 81.66885 |  |  |
| B6 | pc3-n5 | 27.31967 | 26.4516 | 4.513439 | 80.84724 |  |  |
| B7 | pc3-n5 | 33.390419 | 26.4516 | 4.513439 | 85.28392 |  |  |
| B8 | pc3-n5 | 23.712324 | 26.4516 | 4.513439 | 81.50452 |  |  |
| B9 | pc3-n5 | 27.834856 | 26.4516 | 4.513439 | 82.65477 |  |  |
| B10 | pc3-n5 | 21.198019 | 26.4516 | 4.513439 | 81.01156 |  |  |
| B11 | pc3-n5 | 32.271721 | 26.4516 | 4.513439 | 85.44824 |  |  |
| B12 | pc3-n5 | 27.413103 | 26.4516 | 4.513439 | 83.47638 |  |  |
| C1 | pc3-n5 | 28.965107 | 26.4516 | 4.513439 | 78.38242 |  |  |
| C2 | pc3-n5 | 36.743649 | 26.4516 | 4.513439 | 86.10553 |  |  |
| C3 | pc3-n5 | 24.502626 | 26.4516 | 4.513439 | 80.68291 |  |  |
| C4 | pc3-n5 | 27.100269 | 26.4516 | 4.513439 | 85.94121 |  |  |
| C5 | pc3-n5 | 26.781015 | 26.4516 | 4.513439 | 81.3402 |  |  |
| C6 | pc3-n5 | 25.735472 | 26.4516 | 4.513439 | 80.84724 |  |  |
| C7 | pc3-n5 | 27.178322 | 26.4516 | 4.513439 | 80.02563 |  |  |
| C8 | pc3-n5 | 25.169968 | 26.4516 | 4.513439 | 78.87537 |  |  |
| C9 | pc3-n5 | 18.991707 | 26.4516 | 4.513439 | 80.35427 |  |  |
| C10 | pc3-n5 | 23.452417 | 26.4516 | 4.513439 | 75.09598 |  |  |
| C11 | pc3-n5 | 26.683529 | 26.4516 | 4.513439 | 84.13367 |  |  |
| C12 | pc3-n5 | 19.193676 | 26.4516 | 4.513439 | 84.79095 |  |  |
| D1 | pc3-n5 | 20.788816 | 26.4516 | 4.513439 | 85.61256 |  |  |
| D2 | pc3-n5 | 27.233814 | 26.4516 | 4.513439 | 78.54674 |  |  |
| D3 | pc3-n5 | 34.597763 | 26.4516 | 4.513439 | 78.05377 |  |  |
| D4 | pc3-n5 | 33.969784 | 26.4516 | 4.513439 | 78.38242 |  |  |
| D5 | pc3-n5 | 29.146229 | 26.4516 | 4.513439 | 78.71105 |  |  |
| D6 | pc3-n5 | 23.603111 | 26.4516 | 4.513439 | 83.31206 |  |  |
| D7 | pc3-n5 | 28.183775 | 26.4516 | 4.513439 | 84.13367 |  |  |
| D8 | pc3-n5 | 32.793331 | 26.4516 | 4.513439 | 81.50452 |  |  |
| D9 | pc3-n5 | 28.502132 | 26.4516 | 4.513439 | 85.94121 |  |  |
| D10 | pc3-n5 | 26.593094 | 26.4516 | 4.513439 | 81.99748 |  |  |
| D11 | pc3-n5 | 21.866325 | 26.4516 | 4.513439 | 81.99748 |  |  |
| D12 | pc3-n5 | 34.940536 | 26.4516 | 4.513439 | 86.43417 |  |  |
| E1 | pc3-n5 | 25.173378 | 26.4516 | 4.513439 | 75.75327 |  |  |
| E2 | pc3-n5 | 24.516266 | 26.4516 | 4.513439 | 78.38242 |  |  |
| E3 | pc3-n5 | 24.4877 | 26.4516 | 4.513439 | 81.17588 |  |  |
| E4 | pc3-n5 | 23.75104 | 26.4516 | 4.513439 | 75.75327 |  |  |
| E5 | pc3-n5 | 23.15033 | 26.4516 | 4.513439 | 77.39648 |  |  |
| E6 | pc3-n5 | 27.514336 | 26.4516 | 4.513439 | 78.21809 |  |  |
| E7 | pc3-n5 | 38.432018 | 26.4516 | 4.513439 | 88.57035 | 82.81909 | 64.08643 |
| E8 | pc3-n5 | 24.692333 | 26.4516 | 4.513439 | 79.69698 |  |  |
| E9 | pc3-n5 | 23.386808 | 26.4516 | 4.513439 | 79.69698 |  |  |
| E10 | pc3-n5 | 32.494827 | 26.4516 | 4.513439 | 85.28392 |  |  |
| E11 | pc3-n5 | 26.279629 | 26.4516 | 4.513439 | 81.3402 |  |  |
| E12 | pc3-n5 | 30.275291 | 26.4516 | 4.513439 | 84.62663 |  |  |
| F1 | pc3-n5 | 35.911491 | 26.4516 | 4.513439 | 83.80502 |  |  |
| F2 | pc3-n5 | 34.971687 | 26.4516 | 4.513439 | 74.43869 | 83.6407 |  |
| F3 | pc3-n5 | Undetermined | 26.4516 | 4.513439 | 61.95025 |  |  |
| F4 | pc3-n5 | Undetermined | 26.4516 | 4.513439 | 62.27889 |  |  |
| F5 | pc3-n5 | 26.98802 | 26.4516 | 4.513439 | 79.20402 |  |  |
| F6 | pc3-n5 | 25.709787 | 26.4516 | 4.513439 | 74.11005 |  |  |
| F7 | pc3-n5 | 26.683571 | 26.4516 | 4.513439 | 82.32613 |  |  |
| F8 | pc3-n5 | 22.197147 | 26.4516 | 4.513439 | 78.87537 |  |  |
| F9 | pc3-n5 | 23.293633 | 26.4516 | 4.513439 | 82.81909 |  |  |
| F10 | pc3-n5 | 18.705507 | 26.4516 | 4.513439 | 78.05377 |  |  |
| F11 | pc3-n5 | 25.202801 | 26.4516 | 4.513439 | 81.83317 |  |  |
| F12 | pc3-n5 | 22.583954 | 26.4516 | 4.513439 | 84.62663 |  |  |
| G1 | pc3-n5 | 29.821095 | 26.4516 | 4.513439 | 78.05377 |  |  |
| G2 | pc3-n5 | 27.951849 | 26.4516 | 4.513439 | 84.13367 |  |  |
| G3 | pc3-n5 | 23.991098 | 26.4516 | 4.513439 | 78.38242 |  |  |
| G4 | pc3-n5 | 28.875689 | 26.4516 | 4.513439 | 86.76282 |  |  |
| G5 | pc3-n5 | 29.897982 | 26.4516 | 4.513439 | 76.90352 |  |  |
| G6 | pc3-n5 | 27.467213 | 26.4516 | 4.513439 | 78.87537 |  |  |
| G7 | pc3-n5 | 25.925865 | 26.4516 | 4.513439 | 77.39648 |  |  |
| G8 | pc3-n5 | 33.260475 | 26.4516 | 4.513439 | 83.14774 | 65.56532 |  |
| G9 | pc3-n5 | 25.601837 | 26.4516 | 4.513439 | 87.09146 |  |  |
| G10 | pc3-n5 | 29.585083 | 26.4516 | 4.513439 | 85.94121 |  |  |
| G11 | pc3-n5 | 31.123573 | 26.4516 | 4.513439 | 77.56081 |  |  |
| G12 | pc3-n5 | 27.511627 | 26.4516 | 4.513439 | 79.69698 |  |  |
| H1 | pc3-n5 | 18.680174 | 26.4516 | 4.513439 | 84.29799 |  |  |
| H2 | pc3-n5 | 21.835743 | 26.4516 | 4.513439 | 78.05377 |  |  |
| H3 | pc3-n5 | 20.20887 | 26.4516 | 4.513439 | 84.13367 |  |  |
| H4 | pc3-n5 | 24.360853 | 26.4516 | 4.513439 | 76.41055 |  |  |
| H5 | pc3-n5 | 17.641317 | 26.4516 | 4.513439 | 80.35427 |  |  |
| H6 | pc3-n5 | Undetermined | 26.4516 | 4.513439 | 62.44321 |  |  |
| H7 | pc3-n5 | 23.260519 | 26.4516 | 4.513439 | 81.66885 |  |  |
| H8 | pc3-n5 | 22.297234 | 26.4516 | 4.513439 | 81.99748 |  |  |
| H9 | pc3-n5 | 20.879179 | 26.4516 | 4.513439 | 82.32613 |  |  |
| H10 | pc3-n5 | 20.227013 | 26.4516 | 4.513439 | 81.66885 |  |  |
| H11 | pc3-n5 | 21.361105 | 26.4516 | 4.513439 | 81.66885 |  |  |
| H12 | pc3-n5 | 21.856422 | 26.4516 | 4.513439 | 81.66885 |  |  |
